# ARHGAP25: a novel player in the Pathomechanism of allergic contact hypersensitivity

**DOI:** 10.3389/fimmu.2025.1509713

**Published:** 2025-02-26

**Authors:** Domonkos Czárán, Péter Sasvári, Kende Lőrincz, Krisztina Ella, Virág Gellén, Roland Csépányi-Kömi

**Affiliations:** ^1^ Semmelweis University, Department of Physiology, Budapest, Hungary; ^2^ Semmelweis University, Department of Dermatology, Venereology and Dermatooncology, Budapest, Hungary

**Keywords:** ARHGAP25, contact hypersensitivity, allergic contact dermatitis, TNCB, GTPase activating protein (GAP)

## Abstract

**Objective:**

Contact hypersensitivity (CHS), or allergic contact dermatitis (ACD), is an inflammatory skin disorder characterized by an exaggerated allergic reaction to specific haptens. During this delayed-type allergic reaction, the first contact with the allergen initiates the sensitization phase, forming memory T cells. Upon repeated contact with the hapten, the elicitation phase develops, activating mostly macrophages, cytotoxic T cells, and neutrophilic granulocytes. Our group previously demonstrated that the leukocyte-specific GTPase-activating protein ARHGAP25 regulates phagocyte effector functions and is crucial in the pathomechanism of autoantibody-induced arthritis. Here, we investigate its role in the pathogenesis of the more complex inflammatory process of contact hypersensitivity.

**Methods:**

For sensitization, the abdomens of wild-type and ARHGAP25 deficient (KO) mice on a C57BL/6 background, as well as bone marrow chimeric mice, were coated with 3% TNCB (2-chloro-1,3,5-trinitrobenzene) or acetone in the control group. After five days, ears were treated with 1% TNCB for elicitation. Swelling of the ears caused by edema formation was evaluated by measuring the ear thickness. Afterward, ears were harvested, and histological analysis, investigation of leukocyte infiltration, cytokine production, and changes in relevant signaling pathways were carried out. ARHGAP25 expression at the mRNA and protein levels was measured using murine ear and human skin samples.

**Results:**

ARHGAP25 expression increased in human patients suffering from contact dermatitis and in contact hypersensitivity induced in mice. Our data suggest that ARHGAP25 expression is infinitesimal in keratinocytes. In the CHS mouse model, the absence of ARHGAP25 mitigated the severity of inflammation in a leukocyte-dependent manner by reducing the infiltration of phagocytes and cytotoxic T cells. ARHGAP25 altered cytokine composition in the sensitization and elicitation phase of the disease. However, this protein did not affect T cell homing and activation in the sensitization phase.

**Conclusion:**

Our findings suggest that ARHGAP25 is essential in developing contact hypersensitivity by modulating the cytokine environment and leukocyte infiltration. Based on these findings, we propose ARHGAP25 as a promising candidate for future therapeutic approaches and a potential ACD biomarker.

## 1 Introduction

Allergic contact dermatitis (ACD) is a common skin disease that affects approximately 15% of the population worldwide, thus having a substantial socio-economic impact (1). The pathomechanism of ACD is associated with inflammation and is classified as a type IV delayed-type hypersensitivity reaction. Its development can be divided into two distinct phases: In the sensitization phase, the first contact with the allergens, the so-called haptens, results in a pro-inflammatory environment in the skin, in which activation of the innate and adaptive immune cells occurs. Langerhans cells in the epidermis or langerin-dermal dendritic cells in the dermis take up the allergen and present them to naive T cells, creating a memory T cell population (2–4). Although it was demonstrated that neutrophils are also required for the sensitization phase, the underlying mechanism is not yet fully understood (5). The second contact with the allergen initiates the elicitation phase, causing the proliferation and infiltration of these memory T cells (especially CD8+ cells), which in turn produce different cytokines (e.g., IFNγ and IL-1β) recruiting and activating other leukocyte types such as macrophages and neutrophilic granulocytes into the dermis and the epidermis. Even though mainly these leukocytes are responsible for the tissue damage, mast cells, NK cells, and γδ T cells are also recruited to the lesion sites (6, 7). Keratinocytes, crucial non-hematopoietic players in the sensitization and elicitation phase, produce different types of cytokines, taking part in the activation of T cells (8). They contribute to creating a pro-inflammatory milieu but are also targeted by cytotoxic T cells (9).

Recently, we reported that ARHGAP25 is an essential regulatory component of developing autoantibody-induced arthritis (10). This GTPase-activating protein turns off RAC monomeric GTPase-mediated signaling by accelerating its GTPase activity (11, 12). This way, ARHGAP25 regulates phagocyte effector functions such as migration, phagocytosis, and superoxide production (13–15). Although this protein was initially considered leukocyte-specific, its importance has also been described in many tumor cell types even though the expression level of ARHGAP25 in non-hematopoietic cells is usually very low (13, 16–20). Surprisingly, ARHGAP25 expression in fibroblast-like synoviocytes was detected in amounts similar to what is expected in neutrophils (10). In the absence of ARHGAP25, the joint operation of fibroblast-like synoviocytes, macrophages, and neutrophils alters the cytokine environment in the synovium, leading to the reduced infiltration of phagocytes, which results in mitigated inflammation and tissue damage (10).

These findings lead us to investigate ARHGAP25’s role in an immunological model with a complex interplay between leukocytes and non-hematopoietic cells to challenge our current views on ARHGAP25’s leukocyte-selective roles in immune responses. Contact hypersensitivity (CHS) involves orchestrated interactions between T cells, leukocytes of the innate immune system, and keratinocytes (6, 7). It was reported that keratinocyte-specific deletion of RAC resulted in normal epidermis development and hair follicle loss. Furthermore, RAC1-null keratinocytes are sensitized to leukocyte-derived IFN-γ, which causes their altered differentiation (44). RAC1 was also shown to regulate tight junction barrier function, motility, and cell cycle progression in these cells (45, 46). Furthermore, we observed that ACD or CHS increased ARHGAP25 gene expression in humans and mice, supporting the importance of ARHGAP25 in the pathomechanism of these diseases.

Thus, we investigated the role of ARHGAP25 in the well-characterized contact hypersensitivity mouse model induced by 2-chloro-1,3,5-trinitrobenzene (TNCB) (21–23). The first administration of TNCB on the abdominal skin sensitizes the mice via a T cell-dependent manner, while the second challenge on the ear skin elicits contact hypersensitivity (24).

In the present study, we show that total or leukocyte-specific absence of ARHGAP25 reduces ear swelling upon TNCB challenge and suppresses the infiltration of phagocytes and T cells into the inflamed ear tissue. The lack of ARHGAP25 alters the cytokine milieu formed by the sensitization and the elicitation with the allergen.

Our data suggest that ARHGAP25 is involved in developing and causing allergic contact dermatitis by regulating leukocytes, mainly cytotoxic T cells and macrophages. The elevated gene expression observed in human patients confirms the protein’s importance in the disease and even raises the possibility that it could be used as a biomarker for ACD.

## 2 Methods

### 2.1 Human skin tissue sample collection

For research purposes, skin samples were taken from patients diagnosed with dermatitis after informed consent (**Table 1**). Symptomatic and asymptomatic sampling areas were selected and biopsied by a dermatologist. Prior to sampling, the selected skin area was properly disinfected and anesthetized with lidocaine infiltration. A biopsy punch (diameter of the circlular blade: 5 mm) was used to excise the skin samples. After hemostasis, the defect was primarily closed with a simple knotted suture. Immediately after excision, the tissue sample was placed on dry ice in sterile Eppendorf tubes for further processing. The studies involving humans were approved by the Committee of Science and Research Ethics of the Medical Research Council (ETT TUKEB) and approved by the Department of Health Administration of the National Public Health Center of Hungary (Ethical approval number: IV/1707-6/2020/EKU).

**Table 1:** Clinical information of patients included in the study.

	Patient 1.	Patient 2.	Patient 3.
Age (years)	76	52	71
Sex	Female	Female	Male
Medical history	Symptoms of necrobiosis lipoiodica, topical neomycin treatment and subsequent disseminated contact dermatitis development	Atopic dermatitis treated with a fragrance containing cream and subsequent symptoms of disseminated contact dermatitis	Treatment of suspected scabies with a benzyl benzoate preparation and due to this, disseminated contact dermatitis development
Biopsy site	Right lower extremity	Right retroauricular region	Right scapular region
Histology	Spongiotic dermatitis, lymphocyte exocytosis, mixed infiltrate with lymphocytes and eosinophils	Spongiotic dermatitis, lymphocyte exocytosis, mixed infiltrate with lymphocytes and eosinophils	Spongiotic dermatitis, lymphocyte exocytosis, mixed infiltrate with lymphocytes and eosinophils
Serology	Normal IgE level (<100 NE/L)	Elevated IgE level 572 NE/L (<100 NE/L), specific IgE against Birch, Mountain, Hazel pollens, and aspergillus	Elevated IgE level 1688 NE/L (<100 NE/L), no specific IgE

### 2.2 Experimental animals

Age-matched male, wild-type, and Arhgap25 knock-out mice on a C57BL/6 background were used for the experiments. Animals were bred in a conventional animal facility at Semmelweis University in individually sterile ventilated cages (Tecniplast, Buguggiate, Italy) and moved to the conventional (MD) room two weeks before experiments. All measurements conducted using experimental mice followed the EU Directive 2010/63/EU for animal experiments and were approved by the Animal Experimentation Review Board of Semmelweis University and the Government Office for Pest County, Hungary (Ethical approval numbers: PE/EA/1967-7/2017, PE/EA/00284-7/2021, BA/73/00070-2/2020).

To generate Arhgap25^-/-^ bone marrow chimeric mice, WT recipient animals carrying the CD45.1 allele on C57BL/6 genetic background were lethally irradiated with 11 Gy from a ^137^Cs source using a GSM D1 irradiator as described previously (25). Afterward, unfractionated bone marrow cells from femurs and tibias of ARHGAP25^-/-^ donor mice (carrying the CD45.2 allele) were injected into the retro-orbital plexus of recipient animals. The repopulation of the hematopoietic compartment by donor cells was confirmed four weeks after the transplantation. To this end, peripheral blood was drawn and labeled for Ly6G and CD45.1 or CD45.2, and samples were analyzed using a flow cytometer (CytoFLEX, BeckmanCoulter). The ratio of donor-derived cells among neutrophils was calculated, and only mice with over 95% of the cells of donor origin were used in our experiments.

### 2.3 Induction of allergic contact hypersensitivity

The shaved skin on the abdomen of animals was coated with 3% (m/v) 2-chloro-1,3,5-trinitrobenzene (TNCB, Sigma Aldrich) dissolved in acetone (Molar Chemicals), this initiates the sensitization phase of the allergic reaction. Control mice received vehicle (acetone) treatment only at this point, therefore, no sensitization was initiated. After five days, the animals were anesthetized with isoflurane (Baxter), and ear thickness was measured using a microcaliper (Kroeplin) by an observer blinded to the experimental setup. Then, both the previously TNCB-treated and control animals were treated topically on the ears with 1% (m/v) TNCB. Since in the case of control animals, this was the first encounter with the allergen, the sensitization phase begins in them at this point. In the case of the other treatment group, this is the second encounter with TNCB, therefore, the elicitation phase of the allergic reaction is initiated in them. After 24 hours, ear thickness was measured again, animals were sacrificed, and the ears were harvested for further experiments.

### 2.4 Epidermis isolation of mouse ears

One day after the TNCB challenge, mice were sacrificed, the ears were coated with betadine for 5 minutes, washed with distilled water, removed, rewashed, and incubated for 5 minutes in 70% ethanol. Afterward, the ears were washed with distilled water three times and incubated in a 5 mg/ml Dispase II (Sigma Aldrich) enzyme cocktail dissolved in DMEM culture medium (Lonza) for 24 hours at 4°C. Subsequently, the ears’ epidermis was peeled off with two forceps, cleaned off the remaining connective tissue in distilled water, and snap-frozen in liquid nitrogen for further analysis.

### 2.5 Gene expression analysis

Human tissue samples and mouse ear samples were isolated, immediately frozen in liquid nitrogen, and then ground. Samples were lysed using TRI Reagent™ (Invitrogen) and stored at -80°C until RNA preparation. Total RNA was extracted according to the manufacturer’s protocol. cDNA was synthesized using iScript™ gDNA Clear cDNA Synthesis Kit (Bio-Rad) following the manufacturer’s instructions. Relative expression levels of mouse *Arhgap25* and human *ARHGAP25* were measured using a LightCycler^®^ 480 system (Roche) with TaqMan hydrolysis probes (see **Table 2**). mouse *Rplp0* and human *RPLP0* were used as the reference genes. The second derivative maximum method was applied for data analysis using LightCycler^®^ Relative Quantification Software (version 1.5.0.39, Roche).

**Table 2:** Primer and probe sequences for gene expression analysis.

gene		sequence
mouse *Rplp0*	forward	5’-CTCGCTTTCTGGAGGGTGTC-3’
	reverse	5’-AGTCTCCACAGACAATGCCA-3’
	probe	5’FAM-TGCCTCGGTGCCACACTCCA-TAMRA3’
mouse *Arhgap25*	forward	5’-CCTCCTTTGACAGGGACACA-3’
	reverse	5’-CTTTGCCTCATCTGCGTTCA-3’
	probe	5’FAM- ACCTCCGAGACCTGCCAGAGCC -TAMRA3’
human *RPLP0*	forward	5’-TCGTCTTTAAACCCTGCGTG-3’
	reverse	5’-TGTCTGCTCCCACAATGAAAC-3’
	probe	5’FAM- CCCTGTCTTCCCTGGGCATCAC-TAMRA3’
human *ARHGAP25*	forward	5’-TGGCTACTGTGATTGGTGTG-3’
	reverse	5’-GGGTATATCCTTGGACTTGGG-3’
	probe	5’FAM-CGAAGACCCTGCCGTGATCATGAG-TAMRA3’

### 2.6 Histology

Excised ears were fixed using 4% paraformaldehyde (Sigma-Aldrich), treated with xylol (Lach:ner), dehydrated in ethanol, and embedded in paraffin using a Leica EG1150H embedding station. Sections were prepared (8 µm) and stained with hematoxylin and eosin (Leica). Representative images were captured on a Nikon fluorescent microscope with a 20x objective.

### 2.7 Measurement of different leukocyte types in the blood and ears of mice

Twenty-four hours after TNCB treatment of the ears of the animals, 20 μl of blood was collected from the tail of each mouse in phosphate-buffered saline (PBS) containing 5% FBS (Capricorn Scientific) and 0,5% heparin (Teva). These blood samples were divided into two parts of equal volumes: in one whole leukocyte number (with CD45-FITC), myeloid cells (with CD11b-eFluor450), neutrophil granulocytes (with Ly6G-PerCP-Cy5.5) and macrophages (with F4/80-PE), in the other part, leukocytes (CD45-FITC), T cells (CD3-eFluor450) helper T cells (CD4-PerCP-Cy5.5) and cytotoxic T cells (CD8-PE) were labeled (all antibodies were purchased from ThermoFisher Scientific). After washing with PBS, samples were measured with a Cytoflex cytometer (Beckman Coulter), and the number of different leukocytes in 1 µl blood was determined using a multi-step gating strategy (**Supplementary Figure S1**). Parallelly, the removed ears were cut into small pieces, and the connective tissue was digested with 200 µg/mL Liberase enzyme cocktail (Roche) in Hank’s Balanced Salt Solution (HBSS, Cytia) supplemented with 200 mM HEPES (Sigma-Aldrich), leaving the cells intact. Afterward, the samples were filtered with a cell strainer (70 µm pore size, Sigma-Aldrich), centrifuged, and supernatants were saved for cytokine measurements. The sedimented fraction was resuspended in PBS containing 5% FBS, divided into two equal volumes, stained for different leukocyte subtypes, and measured by flow cytometry as described above.

### 2.8 Cytokine measurements

The amount of IL-1β and MIP-2 in the ears was determined by sandwich ELISA kits (R&D systems) from the supernatants of the digested ear samples (see above), according to the instructions provided by the manufacturer. A broad picture of the cytokine profile in the different experimental groups was assessed from pooled samples (from 9 mice per group) using a Mouse Cytokine Array kit (R&D systems) following the protocol provided by the company.

### 2.9 *In vitro* migration assay

Untreated WT and ARHGAP25-deficient mice were sacrificed, their femur and tibia were excised, and bone marrow was washed out. Afterward, polymorphonuclear neutrophils (PMN) were isolated using percoll gradient centrifugation as described previously (26). Transwell inserts (3 µm pore size polycarbonate membrane; Corning) and wells of 24-well tissue culture plates were precoated with 10% FBS and washed with HBSS. For stimulus, saved supernatants of the ear lysate of the different treatment groups of WT and KO animals were used. Supernatants were centrifuged at 12000 RPM for 10 minutes to remove cellular debris, pooled (9 samples per experimental group), and diluted 2-fold with HBSS. Wells were filled with 1 ml of these supernatants, then inserts were placed inside, and were filled with 2x10^5^ neutrophils in 200 µl HBSS. After one-hour incubation at 37°C, plates were spun, inserts were removed, and the number of neutrophils that transmigrated into the wells was determined using an acid phosphatase assay (27, 28).

### 2.10 Measurements of T cell numbers and activation from lymph nodes

Five days after treatment of the shaved abdominal skin of animals with either TNCB or acetone, mice were sacrificed, and the inguinal and axillary lymph nodes were harvested. The lymph nodes were mechanically fragmented in PBS and filtered through cell strainers with 70 µm pore size. Total cell numbers for each mouse were counted in a Bürker chamber, then 200 µl of the samples were stained for 1 hour at 4 °C with the following specific fluorophore-conjugated antibodies: CD3-eF450, CD4-PerCP-Cy5.5, CD8-PE, CD69-APC, CD25-FITC (ThermoFisher Scientific). Single-stained and unstained samples were also prepared for compensation. Afterward, samples were centrifuged, washed 3 times with PBS, and measured with flow cytometry. During evaluation, the number of different T cell types (helper and cytotoxic) in all four lymph nodes and the ratios of activated T cells (CD25 and CD69 positive cells) within the T cell subtypes were determined.

### 2.11 Retroorbital transfer of lymph node-derived cells

Using the remaining lymph node-derived cell samples (see above), 5 million cells were taken out, centrifuged, resuspended in alpha-MEM medium (Capricorn Scientific), and injected into the retroorbital plexus of recipient mice. Our three experimental groups were WT recipient mice receiving cells derived from WT lymph nodes (WT→; WT), *Arhgap25^-/-^* recipient mice receiving cells from WT lymph nodes (WT→; *Arhgap25^-/-^*), and WT recipient mice receiving cells derived from *Arhgap25^-/-^* lymph nodes (*Arhgap25^-/-^* →; WT). In each group, we used lymph nodes from mice sensitized with TNCB on the abdomen. After one hour of the cell transfer, the ear thickness of the recipient animals was measured with a micro caliper, and ears were painted with TNCB as described previously. Twenty-four hours later, ear thickness was measured again, and in each case, ear thickness increase was calculated.

### 2.12 Western blot analysis

Harvested mouse ears, epidermis of ears, or human skin samples were snap-frozen and ground up in liquid nitrogen. They were then lysed in the following solution: 30 mM Na-HEPES, 100 mM NaCl, 1% [w/v] Triton-X-100, 20 mM NaF, 1 mM Na-EGTA, 1 mM Na-EDTA, 100 mM benzamidine, 1% [w/v] aprotinin, 1% [w/v] protease inhibitor cocktail, 1% [w/v] phosphatase inhibitor cocktail, and 1% [w/v] phenylmethylsulfonyl fluoride (pH 7.5), on ice for 10 minutes. The protein concentration of the samples was determined according to Bradford. Subsequently, they were incubated for 5 minutes at 95°C with 4x reducing buffer, and 40 µg of each sample was separated on 4-15% polyacrylamide gradient gels (Bio-Rad). Following separation, the proteins were blotted onto nitrocellulose membranes (Bio-Rad), blocked with EveryBlot Blocking Buffer (Bio-Rad), and incubated with the specified primary antibodies (**Table 3**) overnight at 4°C. After this, the bound primary antibodies were labeled with a horseradish peroxidase-conjugated secondary antibody specific to rabbit IgG (GE Healthcare). After the development of X-ray films (Fujifilm), the membranes were stripped (if necessary) with 2% Sodium dodecyl sulfate (SDS, Sigma-Aldrich) and 0.7% 2-mercaptoethanol (Serva) in PBS at 55°C for 20 minutes, and GAPDH was identified as a loading control (Cell Signaling, 1:5000 dilution). Densitometry was conducted using ImageJ software (ver. 1.53o), and the data was normalized to GAPDH for total protein and to total-MAPK for phospho-MAPK signals.

**Table 3:** Antibodies used for Western blot analysis.

Specificity	Dilution	Manufacturer	Catalog number	RRID
Total-p38 MAPK	1:1000	Cell Signaling	9212S	AB_330713
Total-NF-κB p65	1:1000	Cell Signaling	8242S	AB_10859369
Total-E-cadherin	1:1000	Cell Signaling	#3195	AB_2291471
Total-I-κB	1:1000	Cell Signaling	4812S	AB_10694416
Total-β-catenin	1:500	Cell Signaling	#8480	AB_11127855
Phospho-p38 MAPK	1:1000	Cell Signaling	4511S	AB_2139682
GAPDH	1:5000	Cell Signaling	14C10	AB_561053
ARHGAP25	1:5000	ImmunoGenes	–	–
rabbit IgG (secondary)	1:5000	GE Healthcare	RPN4301	AB_2650489

### 2.13 Statistical analysis

All data were analyzed and plotted using GraphPad Prism 10.0.1 Software. Comparison of experimental groups was carried out by two-way ANOVA with Tukey’s multiple comparison tests or when there were only two groups (in the case of ARHGAP25 expression experiments) unpaired t-test. All *p* values <0.05 were considered statistically significant.

## 3 Results

### 3.1 ARHGAP25 is overexpressed in human allergic contact dermatitis and in TNCB-induced contact hypersensitivity in mice

**Figure 1:**
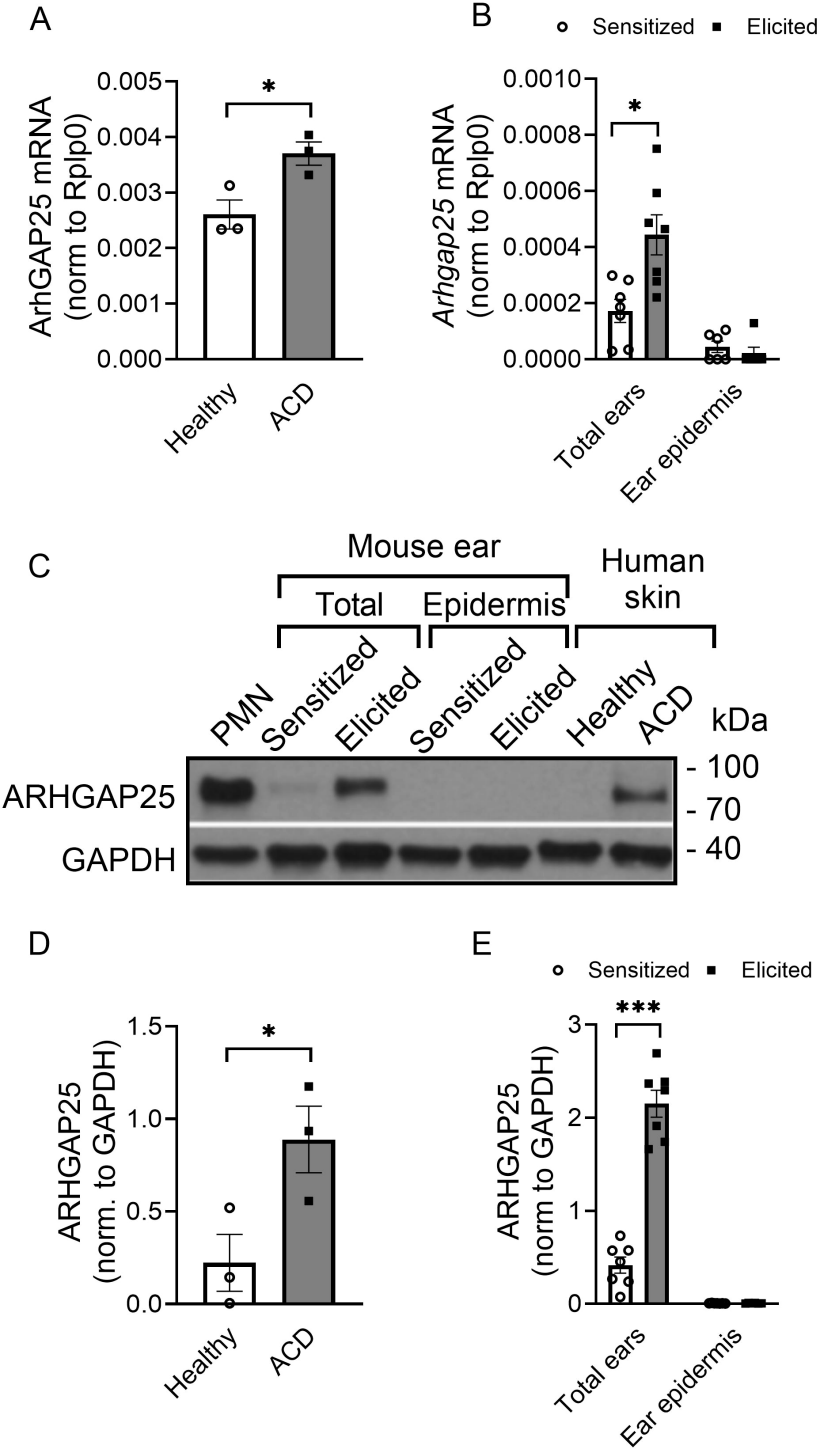
The expression level of ARHGAP25 is elevated in contact hypersensitivity. ARHGAP25 mRNA and protein expression was measured from snap-frozen and lysed human skin samples, mouse ears, and the epidermis of mouse ears. In the qRT-PCR experiments, Rplp0 mRNA was used as a reference gene for normalization. GAPDH proteins was used as a loading control for Western blot experiments. In the case of human samples, skin with allergic contact dermatitis (ACD) had significantly elevated mRNA **(A)** and protein expression of ARHGAP25, compared to healthy skin tissues **(C, D)**. Similarly, in the total ear lysates of mice, allergen treatment resulted in remarkable overexpression both on the mRNA **(B)** and the protein level **(C, E).** On the other hand, neither the mRNA of ARHGAP25 nor the protein itself could be detected in the epidermis of control or allergic mouse ears, suggesting that keratinocytes are not expressing this gene **(B, C, D)**. Mean ± SEM of 7 mice and 3 human samples per group are plotted, *p<0.05, ***p<0.001. PMN, Polymorphonuclear neutrophils; ACD, Allergic contact dermatitis.

It was reported that gene expression of ARHGAP25 can be altered in different types of tumor cells (16–18, 29). We also demonstrated that in terminally differentiated neutrophilic granulocytes, stimulation decreases ARHGAP25 expression (12). However, it is unknown whether complex inflammatory conditions affect ARHGAP25’s expression. Thus, we decided to compare ARHGAP25 expression in skin samples affected by ACD to that of healthy skin areas in self-controlled RT-qPCR and WB experiments. We observed a remarkable elevation of ARHGAP25 mRNA and protein levels in skin areas showing symptoms of ACD compared to the healthy control skin samples collected from the same patients (**Figures 1A, C, D**). The clinical information of patients is indicated in **Table 1**. Next, we decided to use the TNCB-induced contact hypersensitivity model to mimic human ACD in mice. For this, WT animals were treated with either 3% TNCB or, in the case of controls, only the solvent on the abdomen. Five days later, ears were painted with 1% TNCB in both groups, as detailed in the Methods section. RT-qPCR measurements revealed that the *Arhgap25* mRNA and protein levels were increased significantly upon the second exposure to TNCB in the total ear lysates (**Figures 1B, C, E**). According to this, we decided that this murine model is suitable for our experiments, which aim to investigate the role of ARHGAP25 in contact hypersensitivity. Since ARHGAP25 is highly expressed in leukocytes, but lately it was discovered, that certain other cell types express it as well (10, 16, 17, 30), we decided to also measure the expression of this protein in the epidermis of murine ear samples. RT-qPCR and WB experiments revealed no detectable ARHGAP25 in the mouse ear epidermis, neither in the RNA, nor in the protein level, and even the second treatment with the allergen did not increase its expression (**Figures 1B, C, E**). This suggests that keratinocytes do not express ARHGAP25, and the detected signals in human skin, and murine total ear samples is due to the infiltrated leukocytes.

### 3.2 Lacking ARHGAP25 results in decreased ear swelling after repeated TNCB treatment

**Figure 2:**
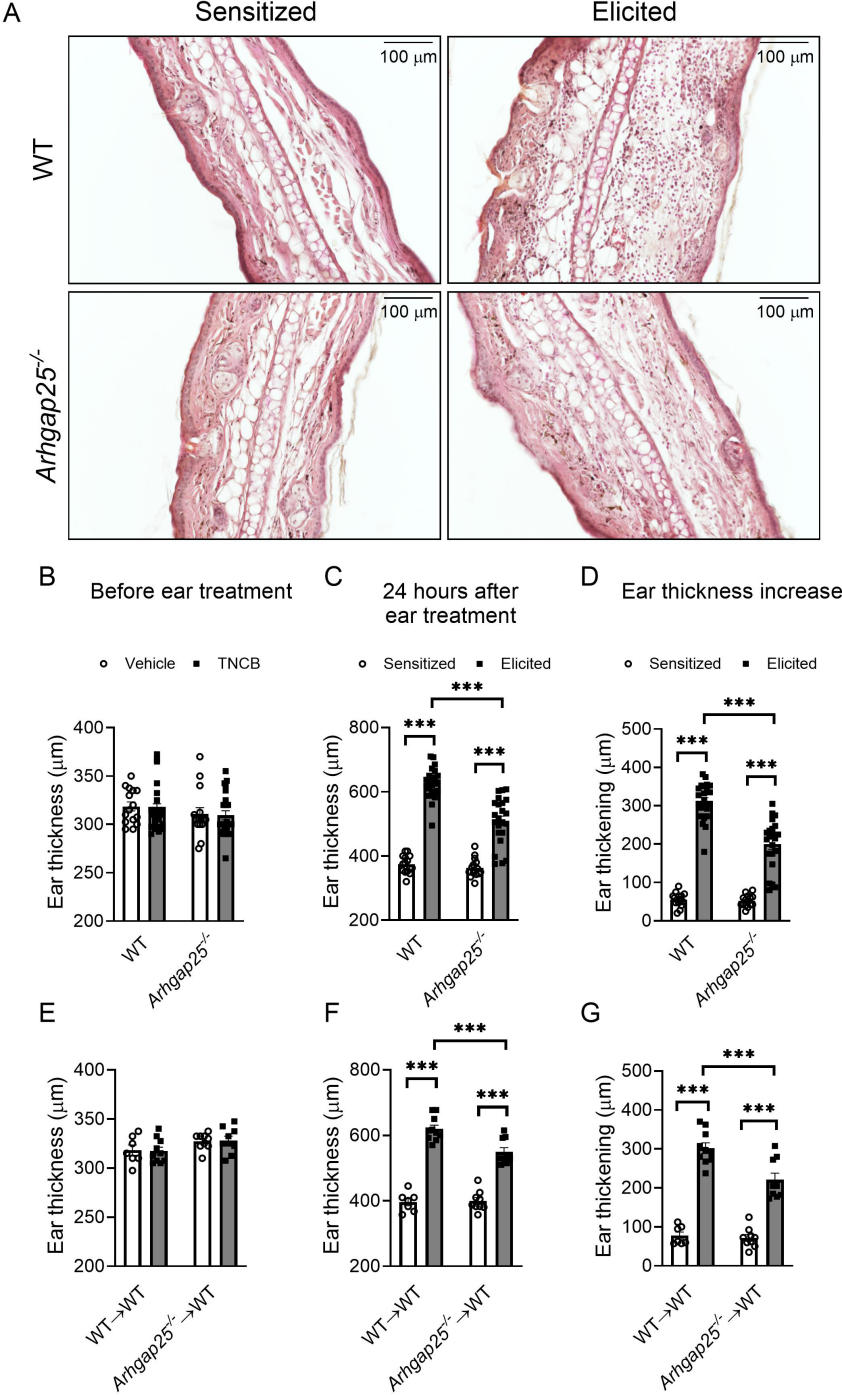
Ear swelling upon contact allergen challenge is mitigated in the absence of ARHGAP25. TNCB treatment resulted in strong leukocyte infiltration, which was reduced in ARHGAP25-deficient animals compared to WT (HE staining, representative picture) **(A)**. Before treatment of the ears with TNCB, there was no difference in ear thickness between KO and WT neither in the sensitized nor in the untreated groups **(B)** Sensitization of the ears with the allergen alone (in the control groups) did not cause a difference in ear thickness between wild-type and ARHGAP25 knockout mice. After elicitation, however, the lack of ARHGAP25 significantly reduced the ear thickening of KO animals **(A, C, D)**. Similarly, in the case of bone marrow chimeric mice carrying ARHGAP25-deficient hematopoietic cells, no ear thickness difference was detected before ear treatment **(E)**, however upon TNCB challenge, reduced ear thickening was measured in KO compared to WT chimeras **(F, G)**. Mean ± SEM of 15-23 animals is plotted in four independent experiments in the case of full WT and KO or 7-10 in two independent experiments in the case of chimeras. ***p<0.001.

To investigate the role of ARHGAP25 in the complex inflammatory process of contact hypersensitivity, allergic reaction was induced in WT, and ARHGAP25 knock-out mice as described previously. One day after the elicitation with TNCB on the ears, histological analysis revealed substantial leukocyte accumulation in WT ears, which was reduced in the case of ARHGAP25 KO animals (**Figure 2A**). Prior to ear treatment, there was no difference in ear thickness between the two genotypes (**Figures 2A, B**). Even after sensitization, measuring and calculating ear thickening revealed no significant difference between WT and KO animals (**Figures 2A, C, D**). However, the second administration of TNCB resulted in a remarkable increase in ear thickening in WT animals, which was significantly lower in KO animals (**Figures 2A, C, D**). We also generated bone marrow chimeric mice to investigate whether only cells with hematopoietic origin are responsible for the observed difference. These animals carry ARHGAP25 knock-out leukocytes on a WT background (*Arhgap25^-/-^* →WT) or, in the case of the control chimeric mice, WT leukocytes on a WT background (WT→WT). Interestingly, similar results were obtained as in the case of regular KO and WT mice. Before the treatment of the ears with the allergen, no difference in ear thickness was detected (**Figure 2E**), and sensitization did not cause any difference between WT and KO chimeras either. After elicitation, however, ear thickening increased in both groups but to a significantly lesser degree in KO chimeric mice (**Figures 2F, G**).

Our results indicate that ARHGAP25 plays a significant role in developing the disease and acts through mostly the hematopoietic cells.

### 3.3 After the second exposure to the contact allergen, the infiltration of phagocytes and T cells is reduced in the ears of *Arhgap25^-/-^* mice

**Figure 3:**
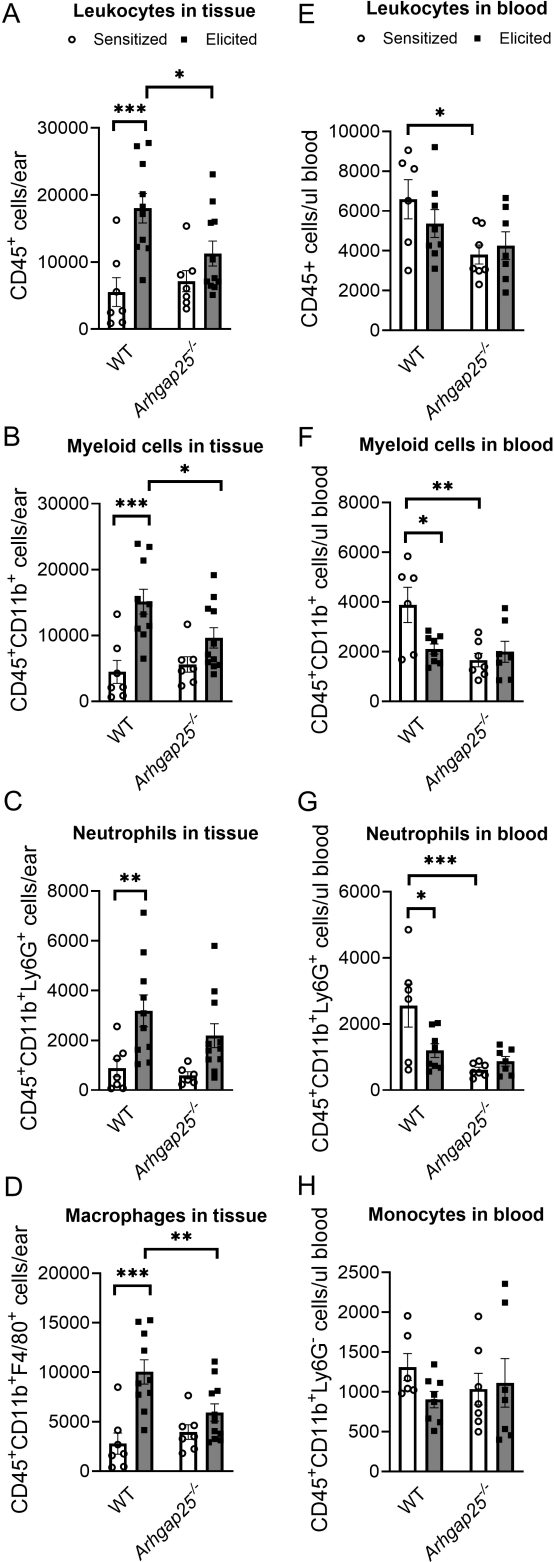
Lacking ARHGAP25 decreased phagocyte recruitment into the inflamed ears. After the TNCB treatment of ears, blood was collected from the tails of wild type and ARHGAP25 knockout mice, ears were digested with Liberase enzyme cocktail, and the numbers of different leukocyte types were determined by flow cytometry, using a multi-step gating strategy. According to our results, in the inflamed ears, leukocytes **(A)** and, in particular, phagocytes of the myeloid lineage **(B)**, mostly macrophages **(D)**, were significantly lower in KO mice compared to WT after second antigen exposure, while neutrophil numbers only slightly decreased **(C)**. In the blood of control animals, the absence of ARHGAP25 resulted in reduced numbers of leukocytes **(E)**, myeloid phagocytes **(F)**, and, most notably, neutrophils compared to WT **(G)**. In contrast, monocyte numbers were not affected **(H)**. Mean ± SEM of 6-11 mice per group in three independent experiments are plotted. *p<0.05, **p<0.01, ***p<0.001.

Next, we measured leucocyte counts from ear tissue and blood to determine whether the observed reduction in inflammatory ear swelling in the case of ARHGAP25-deficient animals is associated with altered leukocyte recruitment. To this end, blood was collected 24 hours after treating the ears with TNCB. Mice were sacrificed, ears were removed, connective tissue was lysed, and cells were labeled with specific antibodies. Flow cytometry analysis revealed that repeated treatment of the WT mice with the allergen caused a significant increase in the number of CD45+ leukocytes, CD45+CD11b+ double-positive myeloid phagocytes, CD45+CD11b+Ly6G+ neutrophils, and CD45+CD11b+F4/80+ macrophages compared to only sensitized animals (**Figures 3A–D**). In the case of the *Arhgap25^-/-^* mice, the cell counts did not differ from WT after sensitization; however, the second TNCB treatment failed to induce a significant increase in the number of any of these leukocyte types (**Figures 3A–D**).

Moreover, the number of CD45+ leukocytes, myeloid cells, and macrophages was significantly reduced in the ear tissue of *Arhgap25^-/-^* mice after elicitation, compared to elicited WT animals (**Figures 3A, B, D**). Analysis of the peripheral blood revealed that the number of WT myeloid cells and neutrophils decreased significantly upon the second TNCB challenge compared to those WT mice, which encountered the allergen only once. This reduction was not present in KO animals (**Figures 3E–G**). Interestingly, the absence of ARHGAP25 decreased the number of leukocytes, myeloid cells, and neutrophils in sensitized animals compared to sensitized WT mice (**Figures 3E–G**), which might be the result of ARHGAP25’s intrinsic effect on leukocyte distribution or its potential role in the sensitization phase too.

At the same time, the number of monocytes did not differ between the WT and KO, either after sensitization or elicitation (**Figure 3H**).

**Figure 4:**
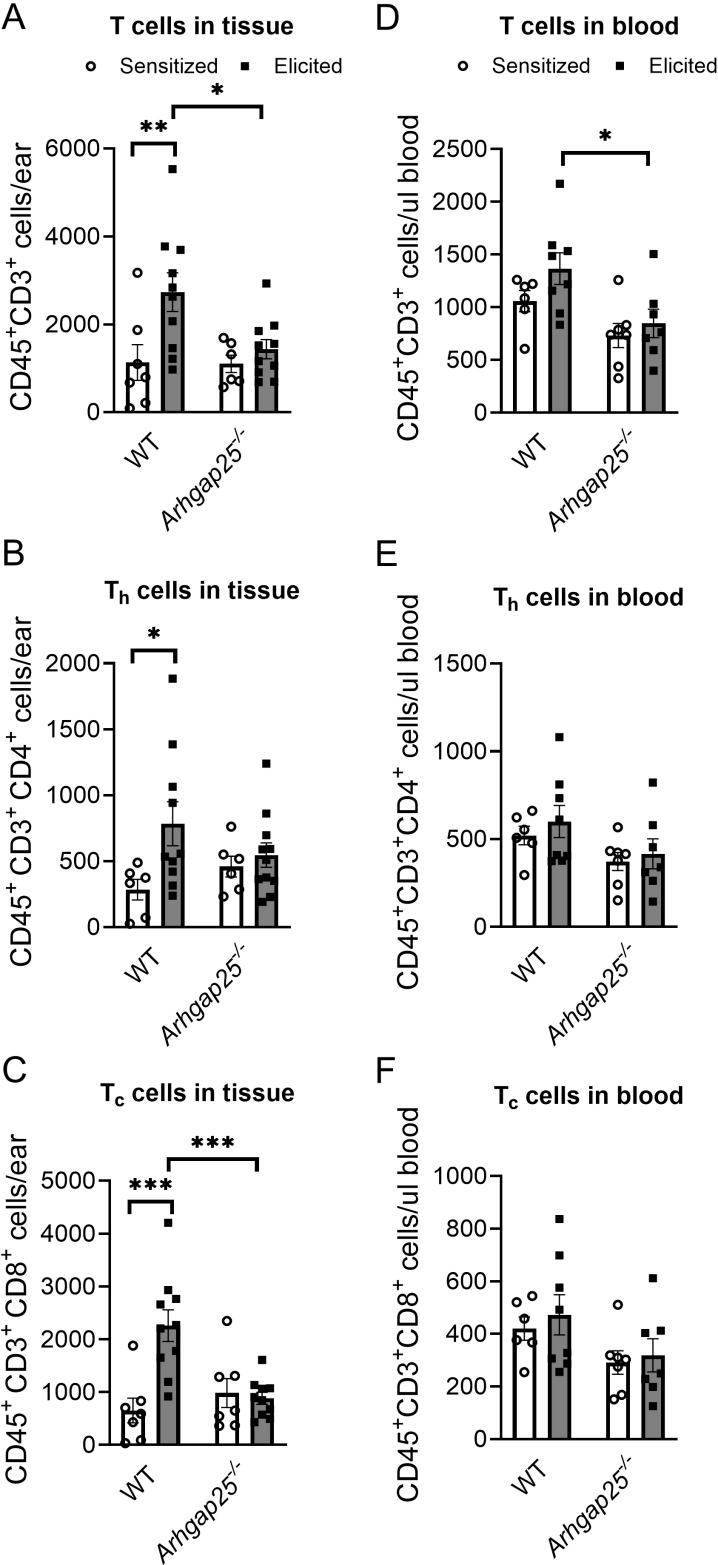
Cytotoxic T-cell infiltration was reduced in ARHGAP25 deficient animals after allergen challenge. Similarly, as in the case of myeloid phagocytes, numbers of different subpopulations of T cells were measured in the blood and the ears of animals after TNCB treatment. In the elicited ears, total T cell recruitment was reduced, cytotoxic T cell numbers decreased **(A, C)**, while helper T cell numbers were unaffected **(B)**. On the other hand, total T cell numbers were reduced in the blood of animals treated with TNCB twice **(D)**, but helper and cytotoxic T cells did not show any alterations between the two genotypes **(E, F)**. Mean ± SEM of 6-11 mice per group in three independent experiments are plotted. *p<0.05, **p<0.01, ***p<0.001.

Helper and cytotoxic T cells are critical players of ACD; thus, we also investigated the number of these cells in the ears and blood. Our measurements revealed that ARHGAP25 deficiency did not alter the number of T cells (CD45+CD3+), helper T cells (CD45+CD3+CD4+), or cytotoxic T cells (CD45+CD3+CD8+) after sensitization. However, the second exposure to the allergen significantly increased these cell counts in the WT but not in *Arhgap25^-/-^* mice. As a result, the difference between elicited WT and KO animals’ T cells and cytotoxic T cell numbers was significant (**Figures 4A–C**). Similarly, the CD45+CD3+ T cell count was decreased in the blood of the *Arhgap25^-/-^* mice after elicitation compared to WT (**Figure 4D**). On the other hand, the number of T cells did not differ in the sensitized animals, just as the number of helper or cytotoxic T cells did not differ, neither in the sensitized nor the elicited animals (**Figures 4D–F**). These data suggest that ARHGAP25 primarily affects the infiltration of effector leukocytes in the elicitation phase of the disease, and the protein is less involved in leukocyte recruitment during sensitization.

### 3.4 In the absence of ARHGAP25, cytokine composition in the allergen-treated ears is altered

**Figure 5:**
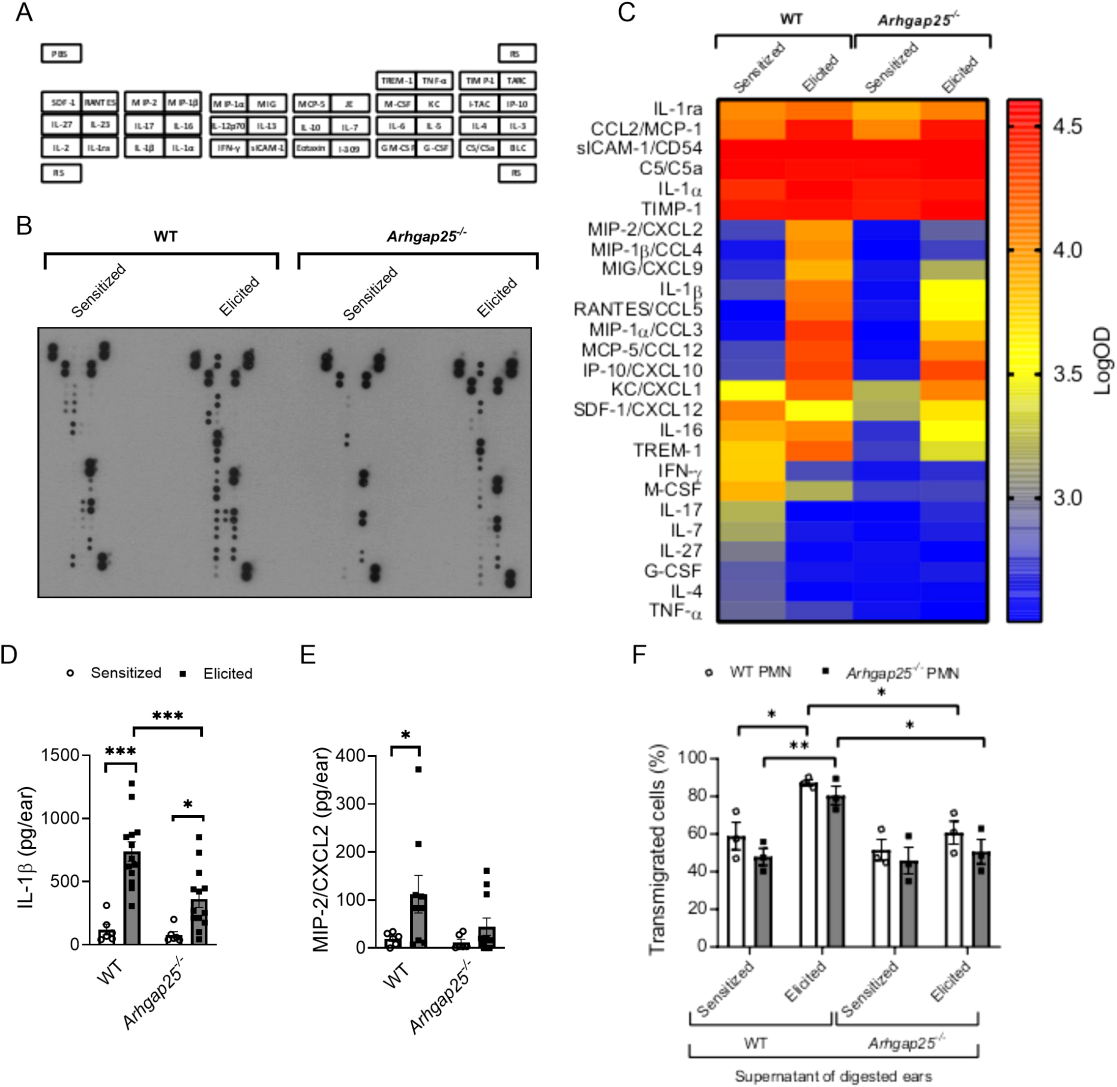
In the absence of ARHGAP25, allergen treatment reduced cytokine production and altered cytokine profile. Pooled samples of the digested ears’ supernatant were used on a cytokine array to detect possible alterations of cytokine profile in the case of ARHGAP25 deficient mice. The map of cytokines immobilized on the membranes in duplicates is shown **(A)**. Representative picture of an x-ray film developed from the array **(B)**. Heat map of integrated pixel density in Logarithmic scale of different cytokines of the array in the experimental groups **(C)**. The concentration of IL-1β and MIP-2 was determined from the supernatants with sandwich ELISA. After TNCB treatment significantly lower IL-1β amount was measured in KO ears compared to WT **(D)**, but only a decreased tendency of MIP-2 amount was measured in the ARHGAP25 deficient ears **(E)**. Transwell assay was conducted using bone marrow-derived neutrophils and pooled, cell-free supernatants of digested ear samples. Both WT and ARHGAP25 KO neutrophils transmigrated to a significantly higher degree when the supernatant of WT elicited ears was added as a stimulus, compared to when the supernatant of WT sensitized ears. Similarly, higher transmigration of both genotypes was measured in the direction of WT elicited supernatant samples compared to KO elicited ones **(F)**. Cytokine array and transwell was measured using pooled samples of 9 animals per experimental group. Mean ± SEM of 6-13 mice per group are plotted in case of the ELISA measurements, and 3 mice per genotype in the case of transwell assay *p<0.05, **p<0.01, ***p<0.001. PMN, Polymorphonuclear neutrophils.

Since the difference in leukocyte infiltration in the case of KO animals could be either the reason or the consequence of an altered cytokine profile, cytokine array and ELISA measurements were conducted. Supernatants of digested ear samples were pooled (9 per experimental group) and used on a mouse cytokine array (**Figures 5A, B**). Densitometric analysis of the array revealed that sICAM-1, C5/C5a, IL-1α, and TIMP-1 were present at a high level in both the sensitized and elicited mice, independently from the genotype. IL-1ra and CCL2 also showed high expression in sensitized animals, but their signal was further increased upon the second exposure to the allergen (**Figures 5A–C**). Expression of most CC and CXC chemokines was low in the sensitized mice, and the second treatment with the allergen increased their level, but to a much higher degree in the WT than in the KO animals. KC (CXCL1) level was in the intermediate range in WT after sensitization, whereas *Arhgap25^-/-^* mice showed lower signals. However, the second allergen challenge increased expression to a similar level in both genotypes. In contrast, the SDF-1 (CXCL12) level decreased in the WT upon the second treatment with the allergen, while it increased slightly in the knock-out. M-CSF expression was in the medium range in the WT before and also after the second treatment with TNCB, whereas it showed lower expression in the KO, in both treatment groups. Interestingly, IFN-γ, IL-17, IL-7, IL-27, and IL-4 signals were decreased in the WT upon elicitation but remained at low levels in the KO in both groups, suggesting an altered cytokine milieu not only in the elicitation, but also in the sensitization phase (**Figures 5A–C**).

Next, we measured the exact concentrations of IL-1β and MIP-2 (CXCL2), which were proven to be influenced by ARHGAP25 in inflammatory conditions (10). The second encounter with the allergen significantly increased the concentration of IL-1β both in WT and KO. However, its amount in the KO mice was considerably lower than in the WT (**Figure 5D**). MIP-2 showed a similar picture; however, we did not observe a significant decrease in the KO compared to the WT after the second encounter with the allergen. It also should be noted that the elicitation did not cause a substantial increase in MIP-2 concentration in the case of KO (**Figure 5E**).

A transwell migration assay was conducted to connect the difference observed in leukocyte infiltration to the altered cytokine composition in the absence of ARHGAP25. With this assay, we could test whether altered cytokine milieu affects neutrophil migration *in vitro*. Bone marrow-derived neutrophils were isolated, and their transmigrating capability toward the cytokine containing pooled, cell-free supernatants obtained from digested ear samples of sensitized or elicited WT and KO mice was measured (9 per experimental group). When we compared the individual effects of each supernatant, we did not observe any difference in the migration of WT and KO neutrophils. However, the supernatant collected from elicited WT animals significantly increased the migration of both WT and KO cells compared to the supernatant of only sensitized WT mice.

Interestingly, the supernatant obtained from the ears of elicited KO mice could not enhance the migration of either WT or KO neutrophils compared to the supernatant of sensitized KO mice. Moreover, transmigration of both WT and ARHGAP25 deficient neutrophils was significantly decreased towards the supernatant of elicited KO, compared to that of elicited WT (**Figure 5F**).

### 3.5 T cell counts and activation are not affected by ARHGAP25 in the draining lymph nodes after sensitization

**Figure 6:**
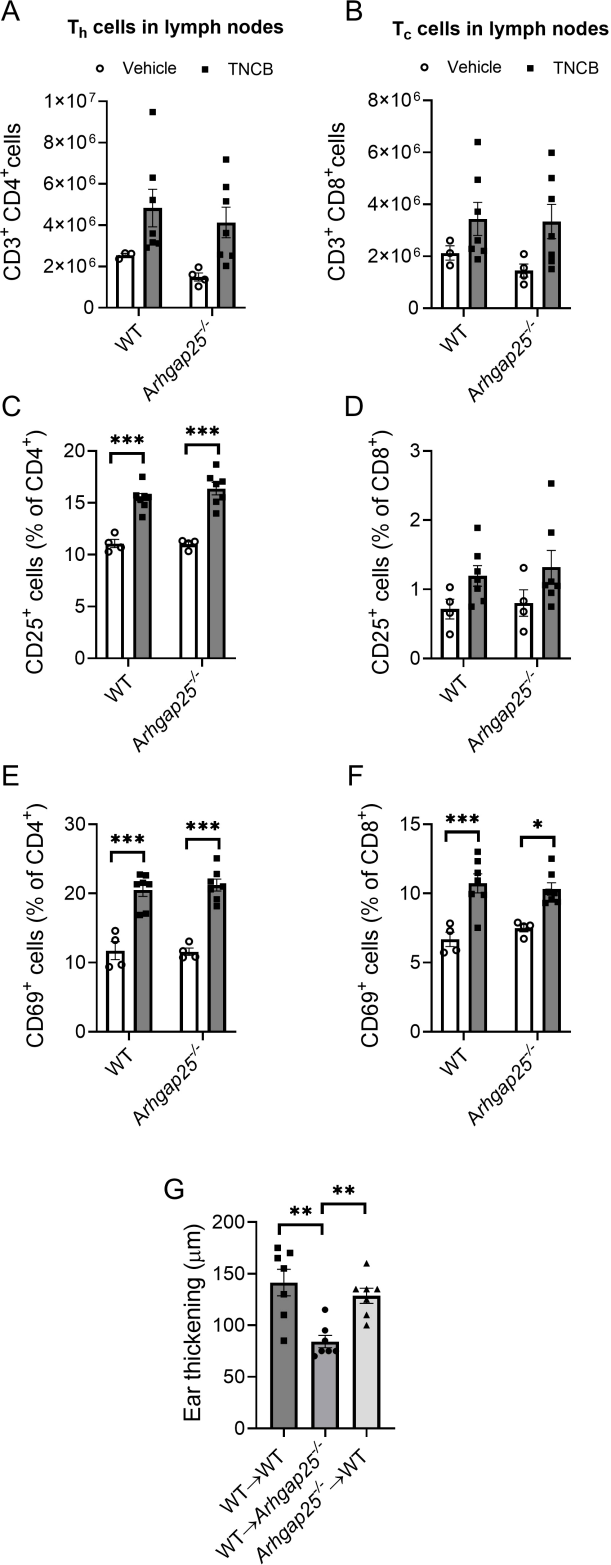
The absence of ARHGAP25 did not affect T cell count and activation in the lymph nodes during sensitization. Inguinal and axillary lymph nodes of mice were excised five days after abdominal allergen or vehicle treatment. After homogenization, the obtained cells were labeled for different T cell-specific (CD3, CD4, and CD8) and T cell activation markers (CD25 and CD69). Flow cytometry revealed that allergen treatment increased the number of T helper cells **(A)** and cytotoxic T cells in the lymph nodes **(B)** independently of the genotype. The ratio of activated, CD25-positive T helper cells significantly increased in the allergen-treated animals compared to vehicle-treated ones, but there was no difference between KO and WT in this regard either **(C)**. In the case of cytotoxic T cells, a similar increase was detectable, although it was not significant **(D)**. Activation was measured through CD69 positivity as well, which significantly increased in both helper **(E)** and cytotoxic T cells **(F)** and was also independent of the genotype. Five million cells derived from lymph nodes of TNCB-treated mice were injected retroorbitally into recipient animals, and the increase in ear thickness was measured after 24 hours. In the case of ARHGAP25 deficient mice receiving WT lymph node-derived cells, ear thickening was significantly lower than WT cells receiving WT and KO cells receiving WT animals **(G)**. Mean ± SEM of 3-7 mice per group in two independent experiments are plotted. *p<0.05, **p<0.01, ***p<0.001.

Since T lymphocytes, especially helper T cells, play a key role in the development of CHS, and the expression of specific cytokines is already altered during sensitization, it is crucial to clarify whether these results could be caused by altered activation or infiltration of ARHGA25-deficient T cells. Thus, we harvested the inguinal and axillary lymph nodes of sensitized WT and *Arhgap25* ^-/-^ mice. Flow cytometric analysis of the lymph node-derived cells revealed that sensitization with TNCB on the abdominal skin significantly increased the total cell number. The helper (CD3^+^CD4^+^) and cytotoxic (CD3^+^CD8^+^) T cell counts in the lymph nodes were also increased in both genotypes; however, the differences were statistically insignificant (**Figures 6A, B**, **Supplementary Figure S2**).

We were also curious whether the activation state of T cells could be altered in ARHGAP25-deficient animals after sensitization. Using flow cytometry, we measured the expression of CD25, as the alpha subunit of the IL-2 receptor is a late activation marker of lymphocytes and regulatory T cells, and the expression of the transmembrane C-type lectin protein CD69, an early activation marker of T cells (31). We found that the ratio of CD25 expressing CD4^+^ helper T cells was significantly increased upon sensitization, while the ratio of CD69 expressing cells was considerably higher within both sensitized CD4^+^ helper and CD8^+^ cytotoxic T cells than the unsensitized control. However, we could not detect significant differences between WT and KO cells (**Figures 6C–F**).

These data suggest that, in the absence of ARHGAP25, neither the number of T cells nor the activation state differs from WT after sensitization in the draining lymph nodes.

Next, we investigated whether the activated, lymph node-derived cells deficient in ARHGAP25 could reduce the severity of CHS on a WT background. Thus, we obtained cells from the inguinal and axillary lymph nodes of sensitized WT and *Arhgap25* ^-/-^ mice and transferred them into resting recipient WT and KO animals (as a sensitization step). After the cell transfer, the ears of the recipient mice were treated with TNCB as elicitation with the allergen, and ear thickness was measured as previously. Surprisingly, we found that ear thickening was reduced significantly only in KO-recipient animals receiving WT lymph node cells (WT→ *Arhgap25^-/-^*). Ear thickening after transferring KO lymph node cells into WT mice (*Arhgap25^-/-^* → WT) did not differ from the wt cell recieving wt recipient (WT→ WT) (**Figure 6G**).

These data suggest that ARHGAP25 is rather involved in the elicitation phase of CHS than in the sensitization phase. Furthermore, it is likely that altered T cell functions, such as reduced activation, are not responsible for the milder inflammation observed in the absence of ARHGAP25 but rather the altered cytokine environment is. Of course, it cannot be ruled out that KO T cells also participate in forming the altered cytokine environment. However, a detailed investigation of the role of ARHGAP25 in T cells is beyond the scope of this project and will be the subject of another publication.

### 3.6 ARHGAP25 affects β-catenin-signaling in the sensitization phase of contact hypersensitivity

**Figure 7:**
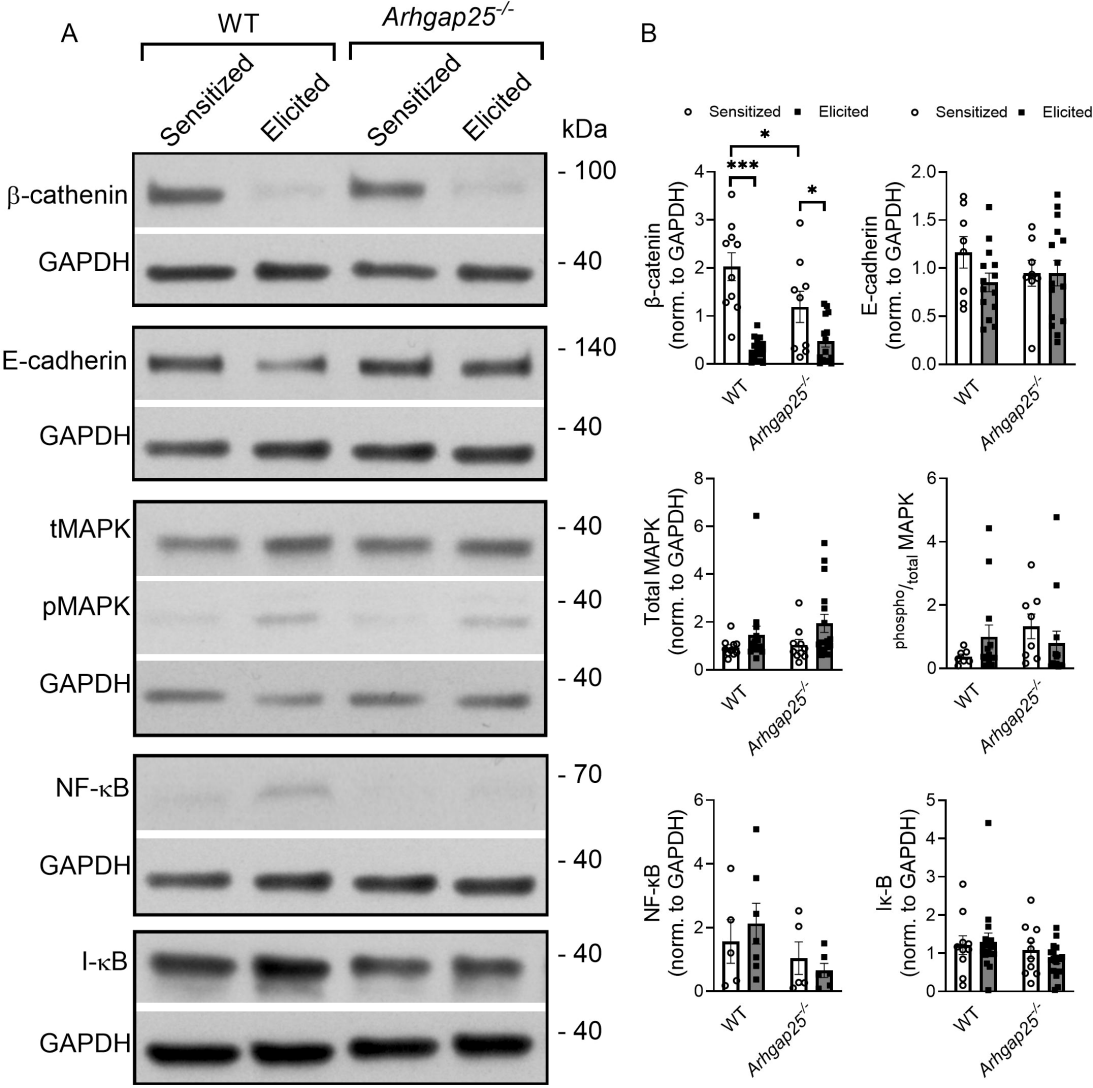
Investigation of the signaling pathways likely affected by ARHGAP25 in allergic dermatitis. Twenty-four hours after treating ears with TNCB, ears were removed, snap-frozen in liquid nitrogen, lysed, and western blot analysis of different signaling proteins was conducted. Representative western blots for the β-catenin, E-cadherin, p38MAPK, NF-κB, and Iκ-B are shown **(A)**. Densitometric evaluation of the Western blots **(B)**. Mean ± SEM of 5-16 mice per group are plotted. *p<0.05; ***p < 0.001.

Next, we set out to investigate the possibly altered signaling pathways by ARHGAP25, which could result in the previously described changes in allergic inflammation. Western blot experiments targeting β-catenin, E-cadherin, p38 MAPK, NF-κB, and I-κB were conducted from whole ear tissue lysates (**Figure 7A**). Densitometric analysis revealed that the second challenge with the allergen significantly reduced β-catenin expression in WT and KO. We found that sensitization alone also resulted in a significant decrease of β-catenin level in the KO compared to WT (**Figure 7B**). However, neither the second challenge with TNCB nor the genotype affected the expression of E-cadherin, total p38MAPK, phospho/total p38MAPK, or I-κB (**Figure 7B**). Although, based on our previous results, we expected that ARHGAP25 would influence NF-κB signaling, we only managed to observe small, non-significant changes: After the second exposure to the allergen, NF-κB expression is reduced slightly in KO mice compared to WT (**Figure 7B**).

## 4 Discussion

An in silico analysis in 2004 discovered ARHGAP25 as a new member of the ARHGAP gene family (32). These proteins encoded by ARHGAP genes regulate the monomeric (or so-called “small”) G proteins of the Rho family. Our research group was the first to clone the full-length ARHGAP25 protein and proved that its GTPase activating effect is specific to RAC, a prominent member of the Rho family (13). In silico data mining, Northern blot, and Western blot experiments revealed that ARHGAP25 is present in all the major leukocyte types. As a phagocyte physiology research group, we investigated its role only in neutrophilic granulocytes. Later, a publication described the regulatory effect of ARHGAP25 on B cells (33), but there is still no data about its role in other immune cells, such as dendritic cells or T lymphocytes.

Meanwhile, more and more publications report ARHGAP25 as an essential regulator of migration and metastasis of tumor cells with non-hematopoietic origin (16–20, 29, 30, 34, 35). Surprisingly, our recent study revealed that, besides leukocytes, ARHGAP25 is highly expressed in fibroblast-like synoviocytes and may be involved in their regulation during the development of serum-transfer arthritis (10). Interestingly, examining skin samples of human patients suffering from allergic contact dermatitis also showed significantly increased expression of ARHGAP25 at the mRNA and protein levels.

These results prompted us to investigate the role of ARHGAP25 in a more complex inflammatory disease model in which neutrophils and macrophages participate together with dendritic cells, mast cells, T cells, and with non-hematopoietic keratinocytes. For this purpose, we chose the 2-chloro-1,3,5-trinitrobenzene (TNCB)-induced contact hypersensitivity mouse model, a well-known and widely used model of human allergic contact dermatitis. The first TNCB treatment sensitizes the animals, and the second encounter with the allergen triggers the inflammatory disease. Consistent with the human results, we observed increased ARHGAP25 expression in the ear tissue samples of TNCB-treated mice (21–23).

Investigating the severity of inflammation, the first TNCB treatment did not result in any significant difference in the thickening of the ears between WT and ARHGAP25-deficient mice. However, the second encounter with the allergen markedly enhanced the ear thickening, a characteristic disease symptom resulting from intense leukocyte infiltration and edema formation. However, this was significantly less pronounced in KO. Repeating the experiments with bone-marrow chimeric mice, in which only the cells with hematopoietic origin are ARHGAP25 knock-out (or WT in the control), and the non-hematopoietic cells are WT, we found similar differences as in the case of the complete knock-out vs WT. This strongly suggests that ARHGAP25 participates in the development of CHS by regulating leukocyte functions and does not directly affect the non-hematopoietic cells, such as keratinocytes. This was further confirmed by our investigations, where we ruled out the presence of ARHGAP25 in the ear epidermis, even after the elicitation phase.

As we described earlier, lacking ARHGAP25 enhances the migration capability, especially the extravasation of leukocytes (10, 14). Based on this, we would expect an increased leukocyte infiltration in the inflamed ears of KO mice. However, similarly to the serum-transfer arthritis model (10), the absence of ARHGAP25 suppressed immune cell infiltration upon the second exposure of TNCB. This difference was especially obvious in the case of macrophages and cytotoxic T cells, perhaps the most important cell types in the elicitation phase of the disease. These results prompted us to investigate the cytokine milieu in the inflamed ears. With the limitations of the cytokine array in mind (being a semi-quantitative method measured from pooled samples), we observed that most of the CC and CXC chemokines’ levels increased as a response to the second contact with the allergen, and this increase was less pronounced in KO.

In contrast, most interleukins showed a different profile. While they decreased after the second encounter with TNCB in the WT groups, the initial elevation after the sensitization was absent in the case of the KO. These data, supplemented with the quantitative analysis of IL-1β and MIP-2 expression providing similar results, suggest that ARHGAP25 regulates the cytokine environment in inflammatory conditions both in the sensitization and the elicitation phase of CHS. *In vitro* transwell assay supported these findings since supernatants obtained from the ear lysate of elicited KO mice could not increase the migration of either WT or KO neutrophils, whereas supernatants collected from digested ears of elicited WT mice resulted in a similar increase in the migration of both WT and KO cells. These data suggest that not the cell-autonomous migration but the cytokine milieu is affected by the lack of ARHGAP25; therefore, this is the likely reason behind the observed difference in the leukocyte recruitment to the inflamed tissue.

Helper T cells are the key players in the sensitization phase of CHS, while cytotoxic T cells are essential effectors of the elicitation phase. The first contact with TNCB resulted in elevated cell counts and increased T cell activation in the inguinal and axillary lymph nodes, proving a successful sensitization. However, lacking ARHGAP25 did not cause any alteration compared to WT. We successfully initiated sensitization in resting recipient animals by transferring lymph node-derived cells from sensitized mice. However, the second encounter with TNCB decreased ear thickening only if KO recipients received WT lymph node-derived cells, compared to when WT animals received WT lymph node cells. Conversely, in WT recipients which received KO cells, no difference was observed. These results confirmed our hypothesis that ARHGAP25 plays a more significant role in elicitation than sensitization and suggest that the T cell homing and activation in the draining lymph nodes after sensitization is not affected by ARHGAP25.

To reveal the molecular mechanisms behind the altered cytokine composition in the absence of ARHGAP25, β-catenin, E-cadherin, MAPK, NF-κB, and IκB were investigated at the protein level in the allergen-treated ears. KEGG analysis suggested that these candidates are likely involved in processes characteristic of CHS (data not shown). In addition, it was reported that overexpression of a dominant negative RAC mutant in HeLa cells ultimately inhibits TNF-induced NF-kB activation (36). Ximei Wu et al. described that RAC1 activation is necessary for the nuclear accumulation of β-catenin (37). RAC is also involved in the β1 integrin-mediated activation of p38 MAPK (38). Finally, Braga et al. showed that in keratinocytes, RAC is necessary to redistribute E-cadherin to cell borders (39). Based on these findings, the possible role of ARHGAP25 through RAC also arises. Interestingly, we found significant differences only in the case of the β-catenin. Its level was significantly decreased after the second exposure to the allergen, both in WT and KO. However, the absence of ARHGAP25 significantly decreased its level after the first encounter with TNCB. These findings are consistent with the results of Jaewoong et al., who described that β-catenin is involved in regulating inflammatory cytokine release upon house dust allergen and showed that its silencing reduces NF-κB activity (40). Our study could not detect any significant differences in the NF-κB protein level; only a downward trend was observed in favor of the KO after the second challenge with TNCB. In our recent paper we identified two new candidates as molecular interactors of ARHGAP25: Syk and 14-3-3 (41) which have been reported to regulate β-catenin (42, 43). Although ARHGAP25 seems to participate predominantly in the elicitation phase, our data indicates that ARHGAP25 regulates the expression of certain cytokines even during sensitization which suggest that ARHGAP25 may take part in the establishment of an initial pro-inflammatory environment as well, probably through the β-catenin pathway. This may further enhance the inflammatory response during elicitation.

Our study has several limitations that should be considered. While the TNCB-induced mouse model is a well-established tool for studying allergic contact dermatitis (ACD), it does not fully replicate human disease. Additionally, using TNCB as an allergen may not represent the diversity of allergens encountered in human ACD. Our focus on ARHGAP25 provides valuable insights, but it needs to capture the broader complexity of ACD pathogenesis, including interactions with other genes, signaling pathways, and immune cells. The molecular mechanisms underlying ARHGAP25’s role also require further clarification. In human studies, the sample size and potential variability related to, e.g., environmental factors could influence the results. Despite this, we can conclude that ARHGAP25 is necessary to develop contact hypersensitivity. This GTPase-activating protein regulates allergic skin inflammation by modulating the cytokine milieu and, through this, leukocyte infiltration. In addition to being a significant player in inflammatory processes and thus may be a therapeutic target, the increase in its expression observed in human patients suffering from ACD suggests that ARHGAP25 might even be used as a biomarker. However, the clinical relevance of ARHGAP25 in human ACD remains to be validated, requiring further translational research.

## Data Availability

The raw data supporting the conclusions of this article will be made available by the authors, without undue reservation.
