# Supplementary material for: ARHGAP25: a novel player in the Pathomechanism of allergic contact hypersensitivity
Source: Front Immunol. 2025 Feb 26;16:1509713. doi: 10.3389/fimmu.2025.1509713 (PMC11896868; doi:10.3389/fimmu.2025.1509713)
Supplement: Supplementary file 1 [file DataSheet1.pdf]

Supplementary Material

ARHGAP25: a Novel Player in the Pathomechanism of Allergic Contact Hypersensitivity.

Domonkos Czárán, Péter Sasvári, Kende Lőrincz, Krisztina Ella, Virág Gellén, Roland Csépányi-Kömi*

***Correspondence:** Roland Csépányi-Kömi: csepanyi-komi.roland@semmelweis.hu

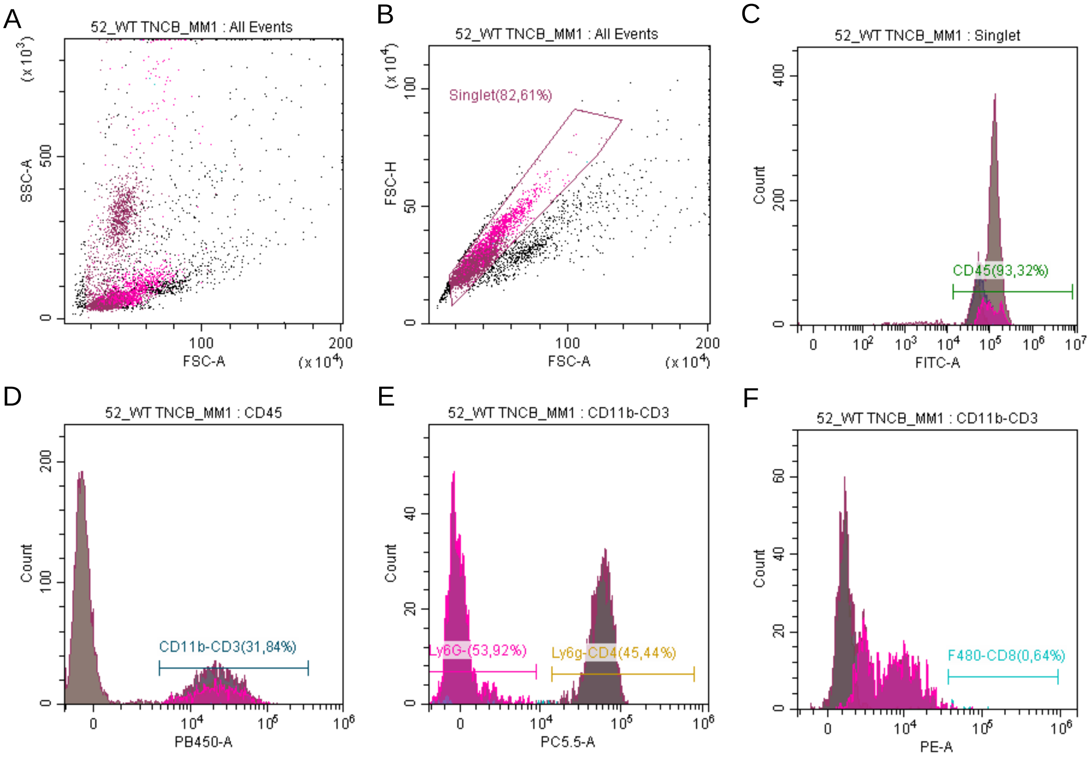

Figure S1. Gating strategy of the flow cytometry measurements. A gating example was obtained through a representative blood sample of a WT, TNCB-elicited mouse. From the total events **(A)**, singlets were selected according to an FSC-A, FSC-H dot-plot **(B)**. From these singlets, CD45 positive events with high FITC fluorescence were gated **(C)**. In this sample, we used the first myeloid-specific master mix (MM1) for staining. CD11b was labeled with a PB450 fluorophore-conjugated antibody, and based on this, CD11b+ cells were selected from the CD45+ cells on the histogram (myeloid leukocytes) **(D)**. From these double-positive events, the Ly6G negative (monocytes) and the Ly6G positive (neutrophils) were selected based on PC5.5 fluorescence **(E)**. Macrophages were also gated from the CD45, CD11b double-positive events based on F4/80-PE positivity (in blood, macrophage numbers are infinitesimal) **(F)**. Compensation with single stained samples was carried out in all cases, and the gates were also constructed according to these samples.

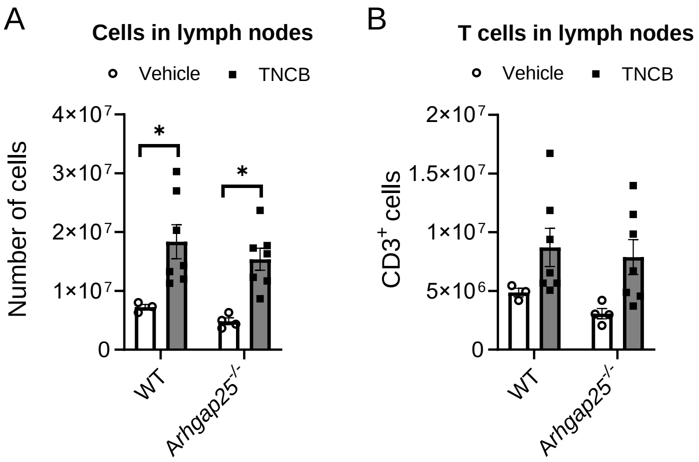

Figure S2. Total cell and T cell counts after sensitization in the draining lymph nodes are not affected by ARHGAP25. Cells obtained from the lymph nodes of TNCB and vehicle-treated animals were counted in a Bürker chamber and labeled with different T cell-specific antibodies as described in the Methods section. Allergen treatment significantly increased total lymph node cell numbers (counted in the Bürker chamber) in both WT and KO animals, but there was no difference between the two genotypes **(A)**. T cell numbers (measured with flow cytometry) were also elevated in TNCB-treated animals. However, this difference was insignificant and independent of the genotype **(B)**. Mean ±SEM of 3-7 mice per group in two independent experiments are plotted. *$p<0.05$.

Original Western blots

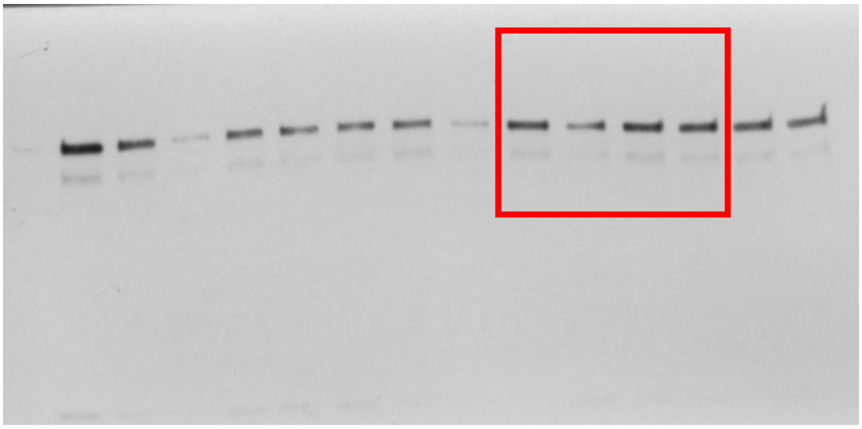

E-cadherin

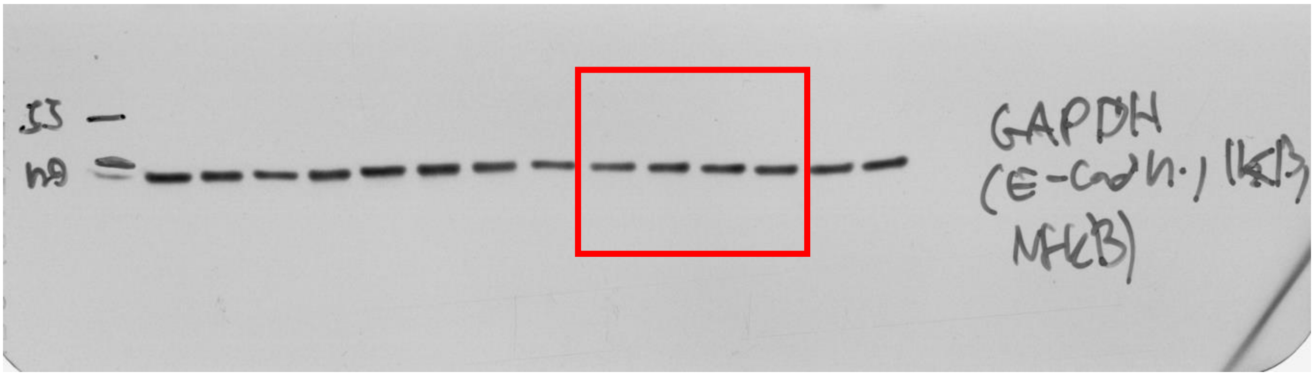

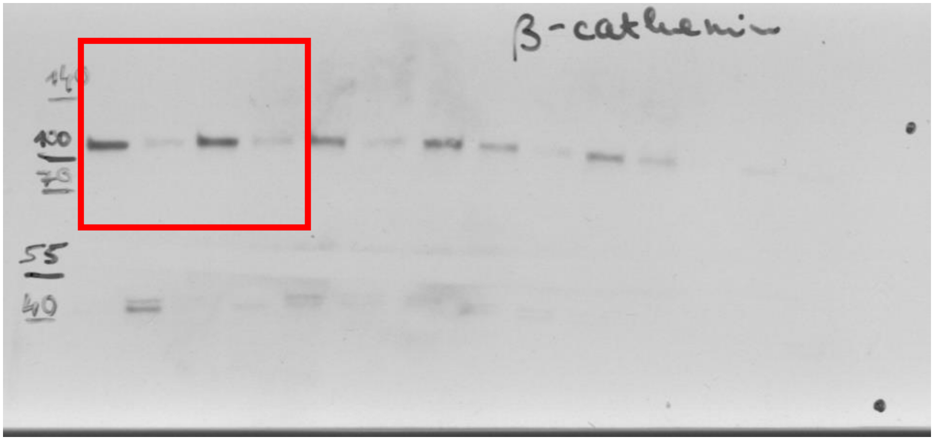

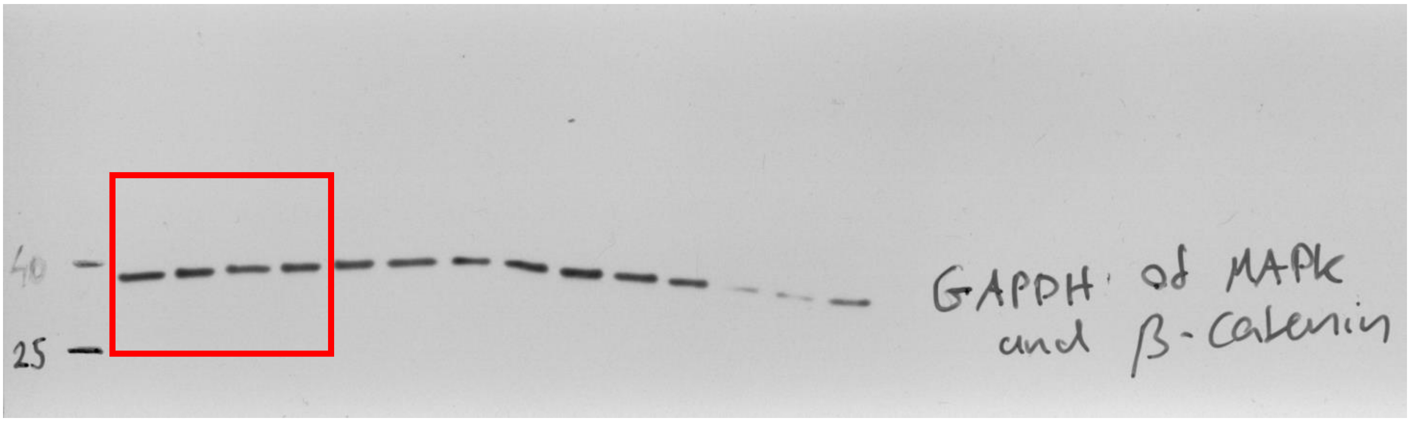

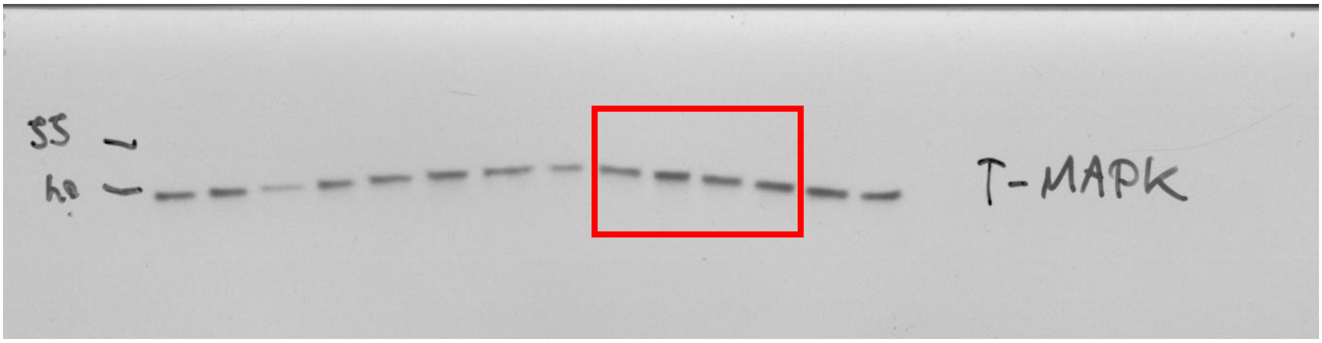

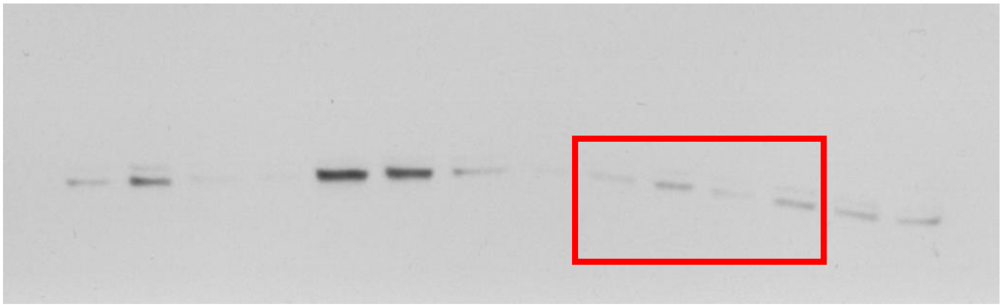

P-MAPK

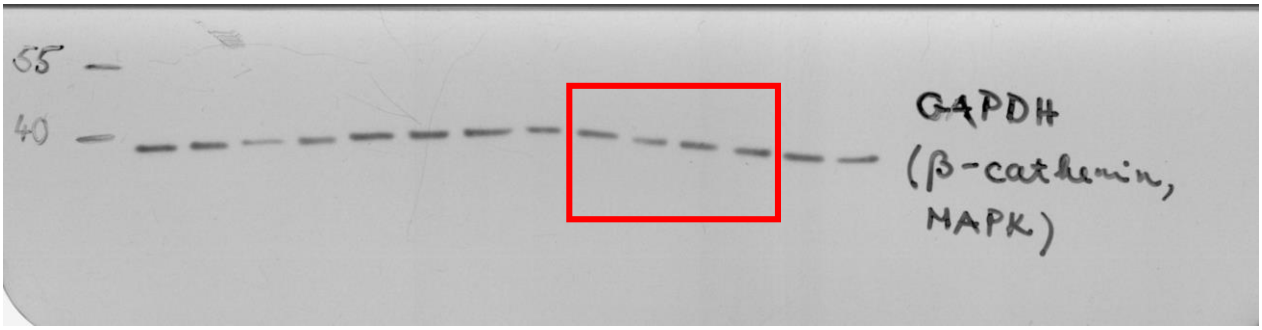

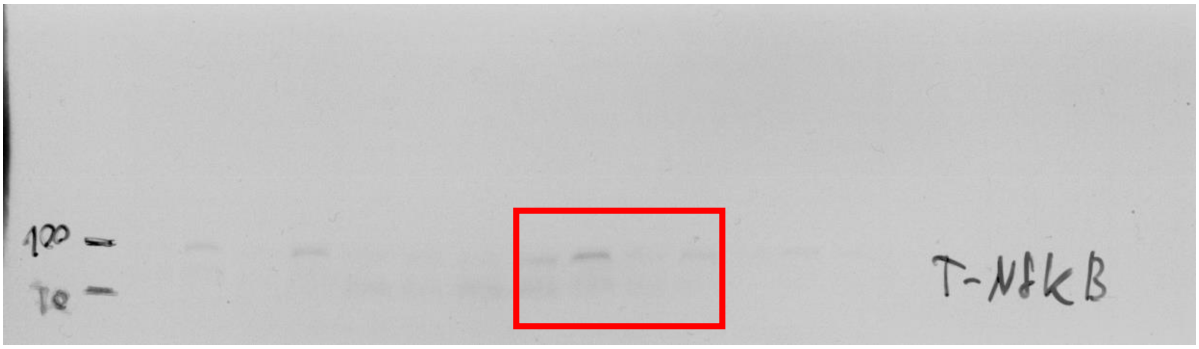

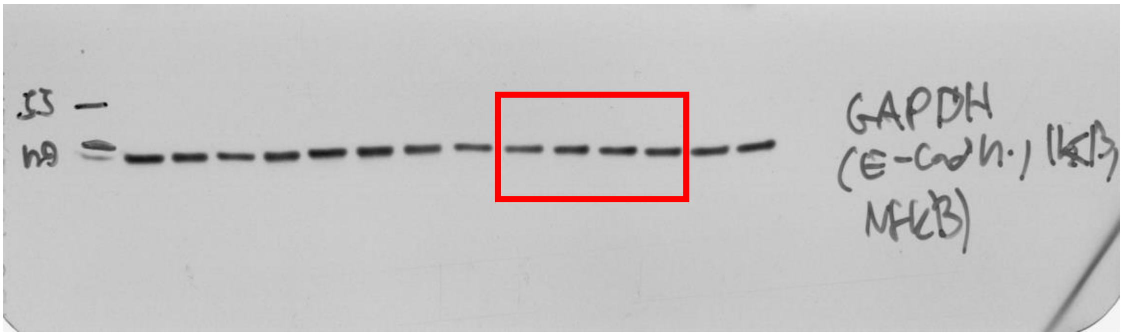

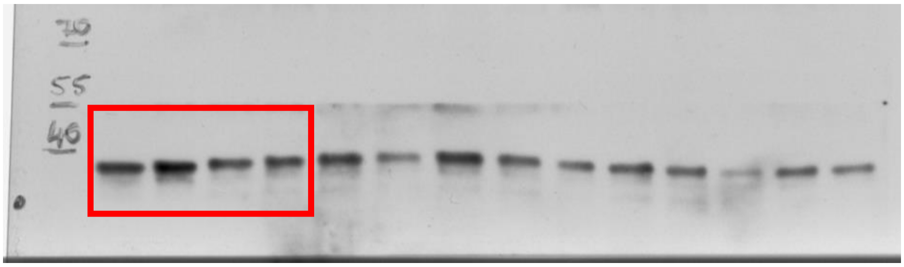

I-κB

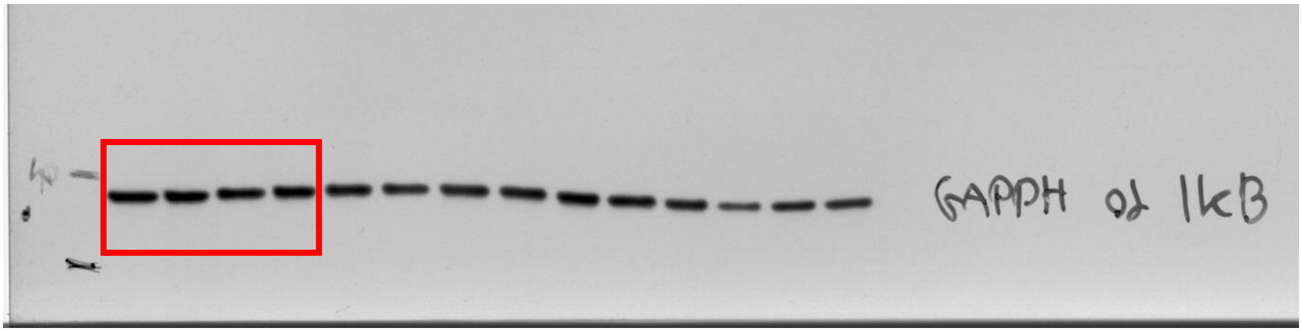

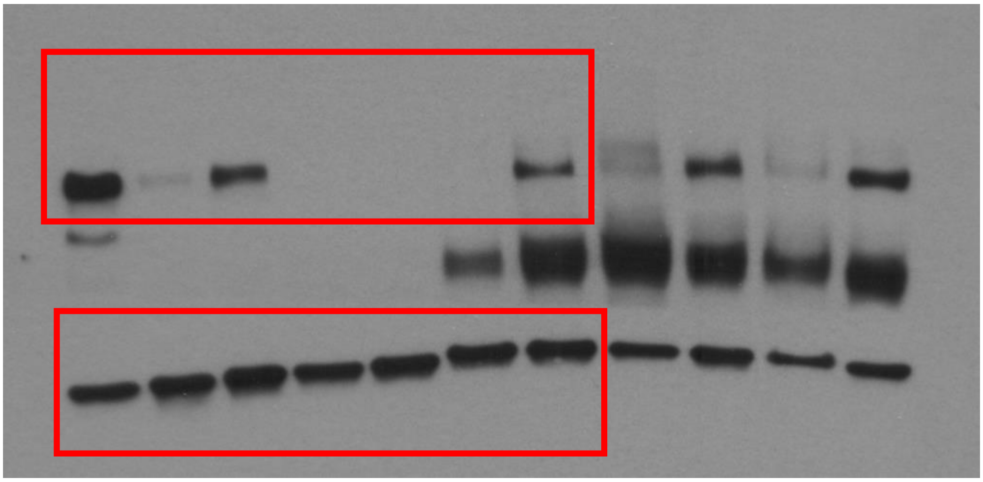

ARHGAP25

GAPDH
